# Antigen Targeting to Dendritic Cells Allows the Identification of a CD4 T-Cell Epitope within an Immunodominant *Trypanosoma cruzi* Antigen

**DOI:** 10.1371/journal.pone.0117778

**Published:** 2015-02-13

**Authors:** Eline V. Rampazo, Kelly N. S. Amorim, Marcio M. Yamamoto, Raquel Hoffmann Panatieri, Mauricio M. Rodrigues, Silvia B. Boscardin

**Affiliations:** 1 Laboratory of Antigen Targeting to Dendritic Cells, Department of Parasitology, Institute of Biomedical Sciences, University of São Paulo, São Paulo, Brazil; 2 CTCMol, Federal University of São Paulo, São Paulo, Brazil; 3 National Institute for Science and Technology in Vaccines, Belo Horizonte, Brazil; Instituto de Ciências Biomédicas / Universidade de São Paulo - USP, BRAZIL

## Abstract

Targeting antigens to dendritic cells (DCs) by using hybrid monoclonal antibodies (mAbs) directed against DC receptors is known to improve activation and support long-lasting T cell responses. In the present work, we used the mAb αDEC205 fused to the *Trypanosoma cruzi* amastigote surface protein 2 (ASP-2) to identify a region of this protein recognized by specific T cells. The hybrid αDEC-ASP2 mAb was successfully generated and preserved its ability to bind the DEC205 receptor. Immunization of BALB/c mice with the recombinant mAb in the presence of polyriboinosinic: polyribocytidylic acid (poly (I:C)) specifically enhanced the number of IFN-γ producing cells and CD4+ T cell proliferation when compared to mice immunized with a mAb without receptor affinity or with the non-targeted ASP-2 protein. The strong immune response induced in mice immunized with the hybrid αDEC-ASP2 mAb allowed us to identify an ASP-2-specific CD4+ T cell epitope recognized by the BALB/c MHCII haplotype. We conclude that targeting parasite antigens to DCs is a useful strategy to enhance T cell mediated immune responses facilitating the identification of new T-cell epitopes.

## Introduction

The potent induction of adaptive immunity provided by DCs is related to at least three innate features these cells possess: (1) they have special mechanisms for antigen capture and processing, (2) once the pathogen is taken up, they migrate to lymphoid organs where the T cells mainly reside, and (3) they rapidly mature in response to a variety of stimuli produced by either the pathogen (pathogen recognition patterns, for example) or by the innate immune response to it (cytokines, innate lymphocytes, immune complexes, among others) [1]. These features can now be harnessed to improve T cell responses against a particular pathogen derived protein. This is accomplished when the sequence of a protein of interest is fused into mAbs that efficiently and specifically target DC receptors in situ [2]. The result is the generation of a hybrid recombinant mAb that, when administered together with an appropriate agonist for DC maturation, improves the efficiency of antigen presentation, inducing CD4 and CD8 T-cell responses besides T-cell help for antibody responses [3–5].

Among the several DC populations present in the steady state [6], there is one that expresses the CD8α chain and a C-type lectin endocytic receptor known as DEC205/CD205 (CD8α^+^DEC205^+^ DCs) [7]. Different antigens were successfully targeted to this particular DC population after their fusion to a mAb known as αDEC205 [3,4,8–10]. The administration of the αDEC205 in the presence of poly (I:C) induced specific Th_1_ cells against proteins expressed by HIV [5,10] and dengue viruses [11], the bacterium *Yersinia pestis* [8,12], and also the protozoan parasite *Leishmania major* [13]. The strength and specificity of such responses have been used to map CD4^+^ T cell immunodominant epitopes derived from different proteins. One CD4^+^ T-cell epitope was described in C57BL/6 mice immunized with αDEC205 fused to ovalbumin while other three were described in C57BL/6 and BALB/c mice immunized with αDEC205 fused to the circumsporozoite protein from *Plasmodium yoelii* [4]. Also, six peptides derived from the HIV gag p24 protein were identified in C57BL/6, BALB/c and C3H/HeJ mice [5]. Similar results were also published for the *Yersinia pestis* protein LcrV [8] and for the *Leishmania major* stress-inducible 1 antigen [13]. These results indicate that antigen targeting to the DEC205^+^ DC population can be used to elicit strong CD4^+^ T cell responses and aid in the search for immunodominant epitopes.

*Trypanosoma cruzi* (*T*. *cruzi*) is an obligate intracellular protozoan parasite and the etiologic agent of Chagas’ disease. Although a significant reduction in transmission was observed in several countries in the last 20 years, Chagas’ disease affects 7 to 8 million people worldwide, mostly in Latin America [14]. The modest prospect of treatment raises the possibility that immunization could be used as an additional weapon to improve disease prevention and treatment efficacy in areas of high transmission.

Mounting evidence in murine models has shown that an effective cellular immune response involving the activation of CD4^+^ and CD8^+^ T cell responses can control parasitemia and improve survival in otherwise lethal infection with *T*. *cruzi* [15–24]. One of *T*. *cruzi* promising antigens, used as a vaccine candidate, is the ASP-2 protein [21]. The intracellular amastigote forms express this antigen, and DNA or recombinant protein vaccination [16,23] and its incorporation into adenoviruses [22] were able to induce strong T cell responses that reduced parasitemia and mediated protection.

In the present study, we genetically fused a 65 kDa portion of the ASP-2 protein to the αDEC205 or to a mAb without receptor affinity (isotype control) and used them to immunize mice in the presence of the toll-like receptor (TLR) 3 and melanoma differentiation-associated gene-5 (MDA5) agonist poly (I:C), as a DC maturation stimulus. In addition, the recombinant protein without any fused antibody was also tested. Our results show that immunization with 2 doses of the fusion αDEC205 mAb elicited a significantly higher number of IFN-γ producing cells and CD4^+^ T cells with proliferative capacity when compared to the non-targeted isotype or the unfused recombinant protein. Moreover, using a few synthetic peptides based on the ASP-2 amino acid sequence, we were able to map a T-cell epitope that is recognized by the BALB/c MHCII haplotype.

## Materials and Methods

### Ethics Statement and Mice

Six- to 8-week-old adult female BALB/c mice were bred under specific- pathogen free conditions at the Isogenic Mouse Facility of the Parasitology Department, University of São Paulo, Brazil. All animals were used according to the Brazilian Council of Animal Experimentation (CONCEA) guidelines. The Institutional Animal Care and Use Committee (CEUA) of the University of São Paulo approved all protocols (approval number 082).

### Plasmid generation

The open reading frame encoding the ASP-2 sequence from the Y strain of *T*. *cruzi* was obtained from plasmid pIgSPclone9 [16]. The sequence equivalent to aminoacids 82 to 694 of the *asp-2* gene [16] was amplified and cloned in frame with the carboxyl terminus of the heavy chain of the mouse DEC-205 (clone NLDC145) and with a mouse isotype control (clone GL-117) mAb (kindly provided by Dr. Michel C. Nussenzweig, The Rockefeller University), as previously described [2]. Amplification was accomplished using the Phusion High Fidelity DNA Polymerase (New England Biolabs) according to the manufacturer’s instructions. The following primers were used: sense (5’-GGCTCGAGGAGTTCGGTAGGTTCCCT CAATGGGTCGATATTTTTG-3’) and anti-sense (5’-GGGCGGCCGCTCAGACCATTTTTAGTTCACCAAC-3’). The underlined sequences represent the *Xho* I and *Not* I restriction endonuclease sites used for the cloning. Plasmids pDEC-ASP2 and pIso-ASP2 were generated and sequenced to confirm the presence of the 65 kDa portion of the ASP-2 protein in frame.

### Expression of hybrid mAbs and recombinant ASP-2 protein

Plasmids containing the heavy chain of the mouse αDEC205 or the isotype control mAbs (pDEC-ASP2 or pIso-ASP2, respectively) and their respective light chains (pDEC kappa or pIso kappa, kindly provided by Dr. Michel C. Nussenzweig, The Rockefeller University) were used to transform DH5 bacteria. Plasmid DNA was purified in large scale with QIAGEN Maxi Prep columns (Qiagen), according to the manufacturer’s instructions.

Transfection was accomplished in human embryonic kidney (HEK) 293T cells (ATCC number CRL-11268) exactly as described in [11]. Fusion mAbs were purified using protein G beads (GE Healthcare), dialyzed against cold PBS and filtered through 0.2 μm membranes (TPP). Each batch was analyzed for integrity in 12% SDS-PAGE gels under reducing conditions. mAb concentration was then estimated by the Bradford assay (Pierce) and the tubes were frozen at -20°C until further use.

The expression and purification of the recombinant ASP-2 protein (rec. ASP2, aminoacids 78 to 694) from the Y strain of *T*. *cruzi* was performed exactly as described in [16].

### Western Blots

Approximately 1 μg of the fusion mAbs or the recombinant protein were run on 12% SDS-PAGE gels under reducing conditions. Gels were either stained with Coomassie Blue (Amresco) or transferred to nitrocellulose membranes (GE Healthcare). Membranes were blocked (PBS-Tween 20 0.05%, non-fat milk 5% and BSA 1%) for 1 hour at room temperature and then incubated with a mAb raised against the ASP-2 protein named K_2_2 [25] in a 1:2,000 dilution. After 3 washes with PBS-T 0.05%, membranes were incubated with protein A labeled with horseradish peroxidase (-HRP) (1:5,000; Invitrogen) for 1 h at room temperature and developed using quimioluminescence (ECL kit, GE Healthcare).

### Binding Assay

Binding assays were performed using CHO cells expressing either the mouse or the human DEC205 receptors, kindly provided by Dr. Michel Nussenzweig (The Rockefeller University), or directly using mouse splenocytes. Either 10, 1 or 0.1 μg/mL of each recombinant mAb were incubated with the cells. Both protocols are described in detail in [11]. For the staining of the two particular DC populations using splenocytes, the following mAbs were used: anti-mouse IgG1-PE (clone A85-1), anti-CD11c-APC (clone HL3), anti-CD49b-biotin (clone DX5), anti-CD19-biotin (clone 1D3), anti-CD3-biotin (clone 145-2C11), anti-MHCII-FITC (clone M5/114.15.2) and anti-CD8-PE-Cy7 (clone 53-6.7). All mAbs were purchased from BD biosciences. Streptavidin-PerCP (BD biosciences) was also used. Thirty thousand events were acquired for the analysis of binding to CHO cells and one million for the analysis of binding to splenocytes using a FACS Canto flow cytometer (BD biosciences). The cells were analyzed using the FlowJo software (version 9.3, Tree Star, San Carlo, CA).

### Immunizations

Groups of 5 mice received 2 doses with a 40-day interval of 5 μg of each hybrid mAb administered intraperitoneally (i.p.) together with 50 μg poly (I:C) (Invivogen). An additional group was also immunized with 2.3 μg of the rec. ASP2 (which corresponds to the amount of ASP-2 protein present on 5 μg of the hybrid mAbs) together with 50 μg poly (I:C). As negative control, a group was immunized with 50 μg poly (I:C) alone.

### Synthetic peptides

Six synthetic peptides (19–21 mer) based on the predicted amino acid sequence of the *asp-2* gene were purchased from New England Peptides (Fitchburg, Massachusetts). They corresponded to amino acids 261 to 380 (Table 1) in the ASP-2 sequence and their purity varied from 78 to 99%.

**Table 1:** Aminoacid sequences and localization within the ASP-2 protein of the peptide pools used in this study.

Pool Number	Peptide Sequence	Amino acid position based on the complete ASP-2 sequence
1	DVVTAGGSGIVMQNDTLVFP	261–280
	LMVNGQNYPFSSITYSTDKG	281–300
2	NTWVFPEGISPVGCLDPRI	301–319
	TEWETGQILMIVQCKDDQSV	320–339
3	FESRDMGKTWTEAIGTLSGV	340–359
	WVMSQPGVRLYKIFRVGALIT	360–380

### Spleen cell isolation and IFN-γ ELISPOT

Mice were euthanized 15 days after the second immunization and spleens were removed aseptically. Bulk splenocytes were obtained in suspension and washed once with RPMI 1640 (Sigma). Red blood cells were lysed with 1 mL ACK solution (150 mM NH_3_Cl, 10mM KHCO_3_, 0.1 mM EDTA) per spleen for 2 minutes at room temperature. After two additional washes with RPMI 1640, splenocytes were resuspended in R10 (RPMI supplemented with 10% of fetal bovine serum (GIBCO), 2 mM L-glutamine (GIBCO), 10 mM Hepes (GIBCO), 1 mM sodium pyruvate (GIBCO), 1% vol/vol non-essential aminoacid solution (GIBCO), 1% vol/vol vitamin solution (GIBCO), 20 μg/mL of ciprobacter (Isofarma, Brazil) and 5x10^–5^ M 2-mercaptoetanol (GIBCO)). Cell viability was evaluated using 0,1% Trypan Blue exclusion dye (GIBCO) and cell concentration was estimated using a hemocytometer.

ELISPOT assays for the detection of IFN- producing splenocytes were performed using the Ready-SET-Go kit (eBioscience), according to the manufacturer’s instructions. Three hundred thousand bulk splenocytes were incubated with 10 μg/mL of rec. ASP2 or ovalbumin (as control), and with 5 μg/mL of the peptide pools or individual peptides. Spots were visualized with the AEC kit (BD biosciences) and counted using an automated stereomicroscope (KS ELISPOT, Zeiss, Oberkochem, Germany). The number of IFN-γ producing cells/ 10^6^ splenocytes was calculated after subtracting the negative control values (number of cells in the wells containing medium only).

### CFSE-based proliferation assay

Three hundred thousand splenocytes from immunized mice were assayed for their ability to proliferate in vitro after stimulation with 1 μg/mL of the rec. ASP2 protein, ovalbumin or individual peptides, using the CFSE dilution based proliferation assay exactly as described in [26].

### Data Analysis

One-way ANOVA followed by Tukey’s honestly significantly different (HSD) test were used to calculate statistical significance (p-values). Prism 5 software (GraphPad Software Inc, LA Jolla, CA) was used for all tests and differences with p≤0.05 were considered statistically significant.

## Results

### Generation of mAbs fused to the ASP-2 protein

**Fig 1:**
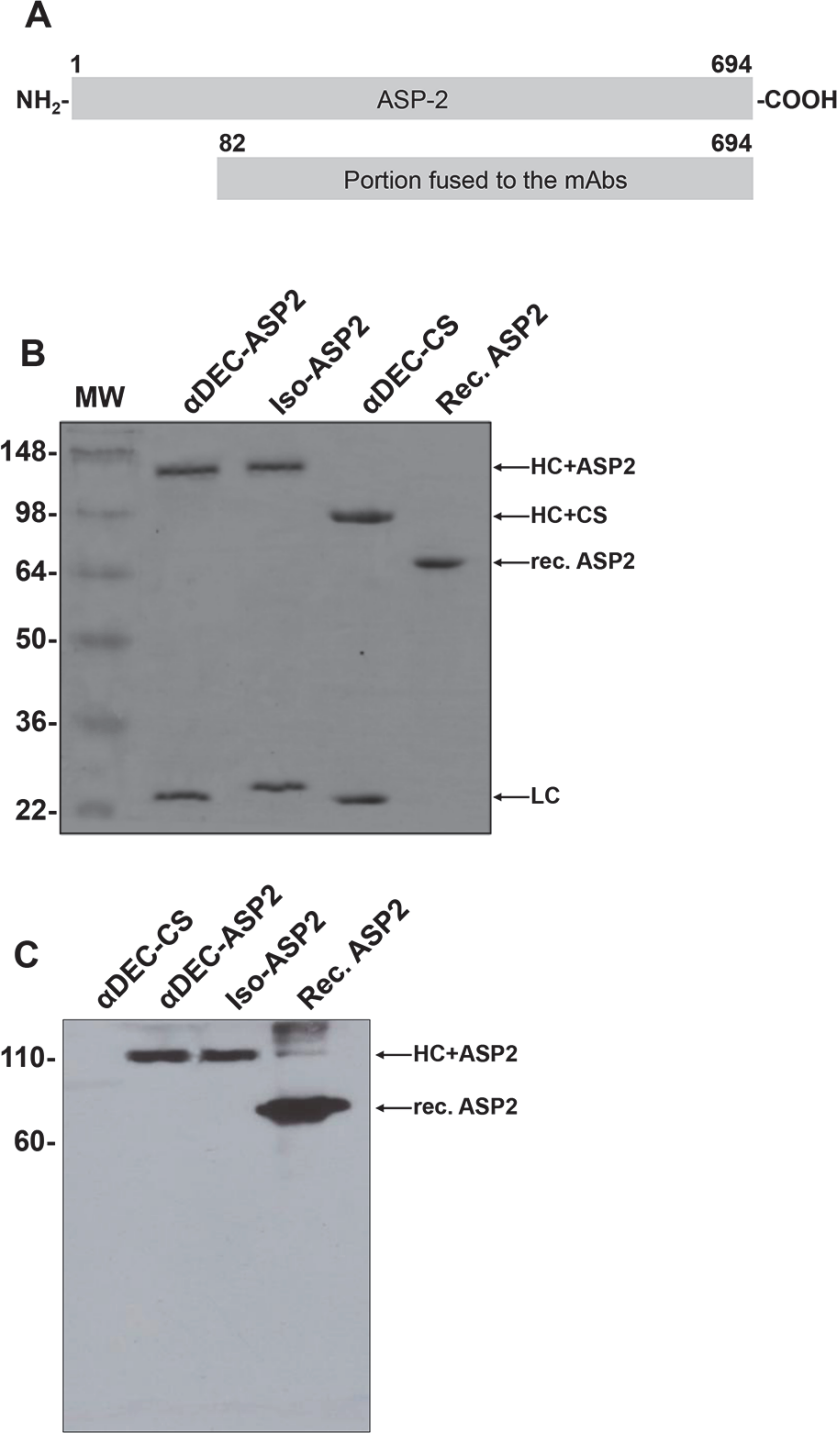
Production and characterization of hybrid mAbs. (A) Schematic representation of the full length ASP-2 protein and of the portion fused to the αDEC205 and Iso mAbs. (B) Approximately 1 μg of each mAb or recombinant protein were separated under reduced conditions. Gel was stained with Coomassie Blue dye. The heavy (HC) and light (LC) chains of αDEC-ASP2, Iso-ASP2 or αDEC-CS (used as control) and rec. ASP2 protein are shown. (C) Western blotting using the same concentration as in (B) and the anti-ASP-2 mAb K_2_2. MW, molecular weight marker in kDa.

For the generation of the hybrid DEC-ASP2 or Iso-ASP2 mAbs, we fused the open reading frames of the αDEC205 or isotype control heavy chains to amino acids 82 to 694 of the ASP-2 protein (Fig. 1A). Fig. 1B shows that heavy and light chains of the purified mAbs had the expected electrophoretic motilities (∼120 and 25 kDa, respectively). As additional controls, we used the mAb DEC-CS that contains the *Plasmodium yoelii* CS protein [4] and the non-fused rec. ASP2 protein [16]. Both controls showed electrophoretic behavior compatible with the expected molecular weights (∼85 and 25 kDa for DEC-CS and 65 kDa for the recombinant protein). Fig. 1C shows that a specific anti-ASP2 mAb [25] recognized the heavy chains of DEC-ASP2 and Iso-ASP2 as well as the rec. ASP2 protein. Altogether these results suggest that the fusion of ASP-2 protein to the mAbs (αDEC205 or isotype control) did not change the conformation of the antigen.

### The DEC-ASP2 mAb binds specifically to cells expressing the DEC205 receptor

**Fig 2:**
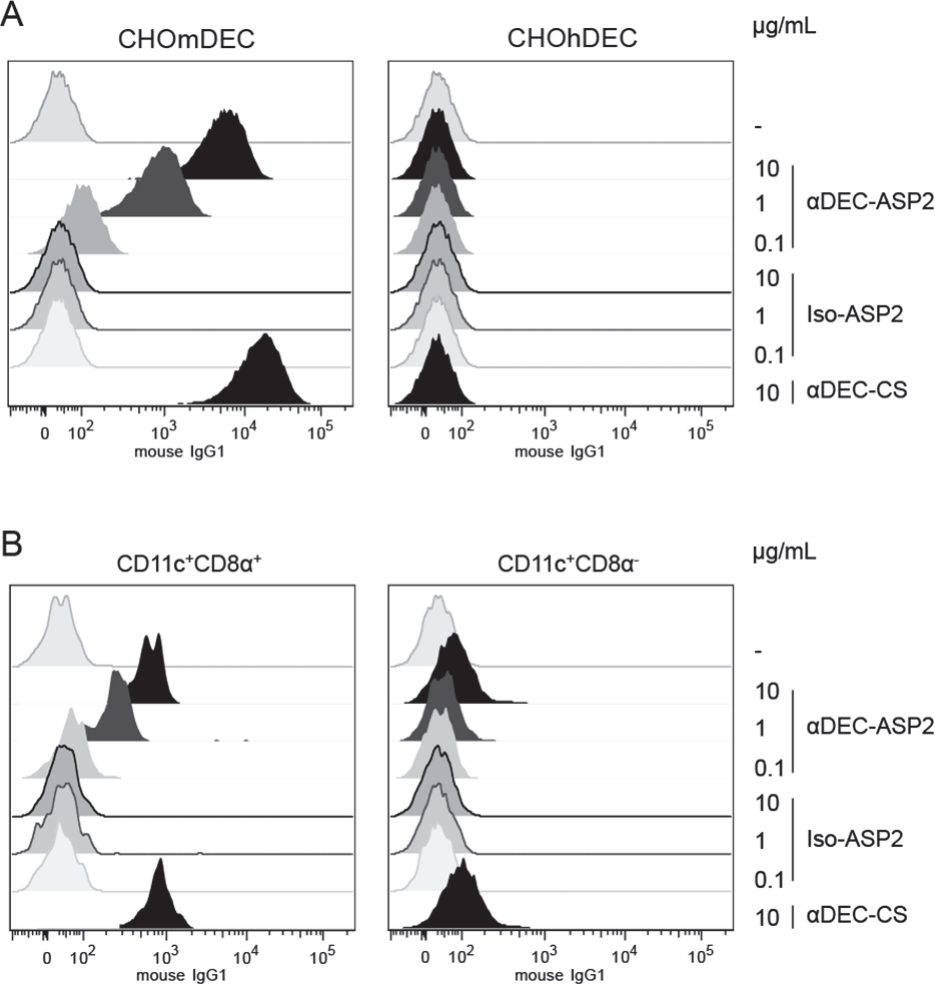
The hybrid DEC-ASP2 mAb binds to the murine DEC205 receptor. (A) 10^5^ CHO cells expressing either the mouse DEC205 (left panel) or the human DEC205 (right panel) receptors were incubated on ice with 10, 1 or 0.1 μg/mL of DEC-ASP2 or Iso-ASP2 mAbs. As positive control, 10 μg/mL of the DEC-CS mAb was used. Binding was detected on 30.000 cells using an anti-mouse IgG1 antibody. One experiment representative of three is shown. (B) 5x10^6^ splenocytes were incubated on ice with the same concentrations of the hybrid mAbs described in (A). S1 Fig. shows the gating strategy for the CD11c^+^CD8^+^ (DEC205 rich population) or the CD11c^+^CD8^-^ DC populations. Binding was detected on 10^6^ cells using an anti-mouse IgG1 antibody. One experiment representative of two is shown.

The αDEC-ASP2 mAb was able to bind specifically and in a dose dependent manner to the mouse DEC205 receptor when incubated with CHO cells expressing it (Fig. 2A, left panel). On the contrary, when the same fusion mAb was incubated with CHO cells expressing the human DEC205 receptor, no binding was observed (Fig. 2A, right panel). As expected, the Iso-ASP2 did not bind to any receptor (Fig. 2A, both panels). As a positive control we used the βDEC-CS mAb [4] that bound specifically to the mouse DEC205 expressing cells (Fig. 2A, left panel).

In addition, we evaluated the specific binding of the αDEC-ASP2 mAb to the mouse CD11c^+^CD8α^+^ DC population that expresses the DEC205 receptor (Fig. 2B). The cells were gated as depicted in the S1 Fig. Once more, the αDEC-ASP2 mAb bound in a dose dependent manner to the CD11c^+^CD8α^+^ DCs (Fig. 2B, left panel). Similarly to Fig. 2A, the αDEC-CS mAb was used as a positive control. We also checked binding to the CD11c^+^CD8α^-^ DC population. In this case, only about 2.4% of the CD11c^+^CD8α^-^ DCs express the DEC205 receptor [27]. As it is shown in Fig. 2B, right panel, we only observed some degree of binding in the highest αDEC-ASP2 mAb concentration (10 μg/mL). The same was observed for the αDEC-CS mAb. As expected, the Iso-ASP2 mAb did not bind to any of the DC populations analyzed (Fig. 2B, both panels). We conclude that the addition of a portion of the ASP-2 protein to the C-terminus of the αDEC205 mAb did not alter the conformation in a way that could compromise its binding to the DEC205 receptor.

### Analysis of the cellular immune response in mice immunized with the hybrid mAbs in the presence of poly (I:C)

**Fig 3:**
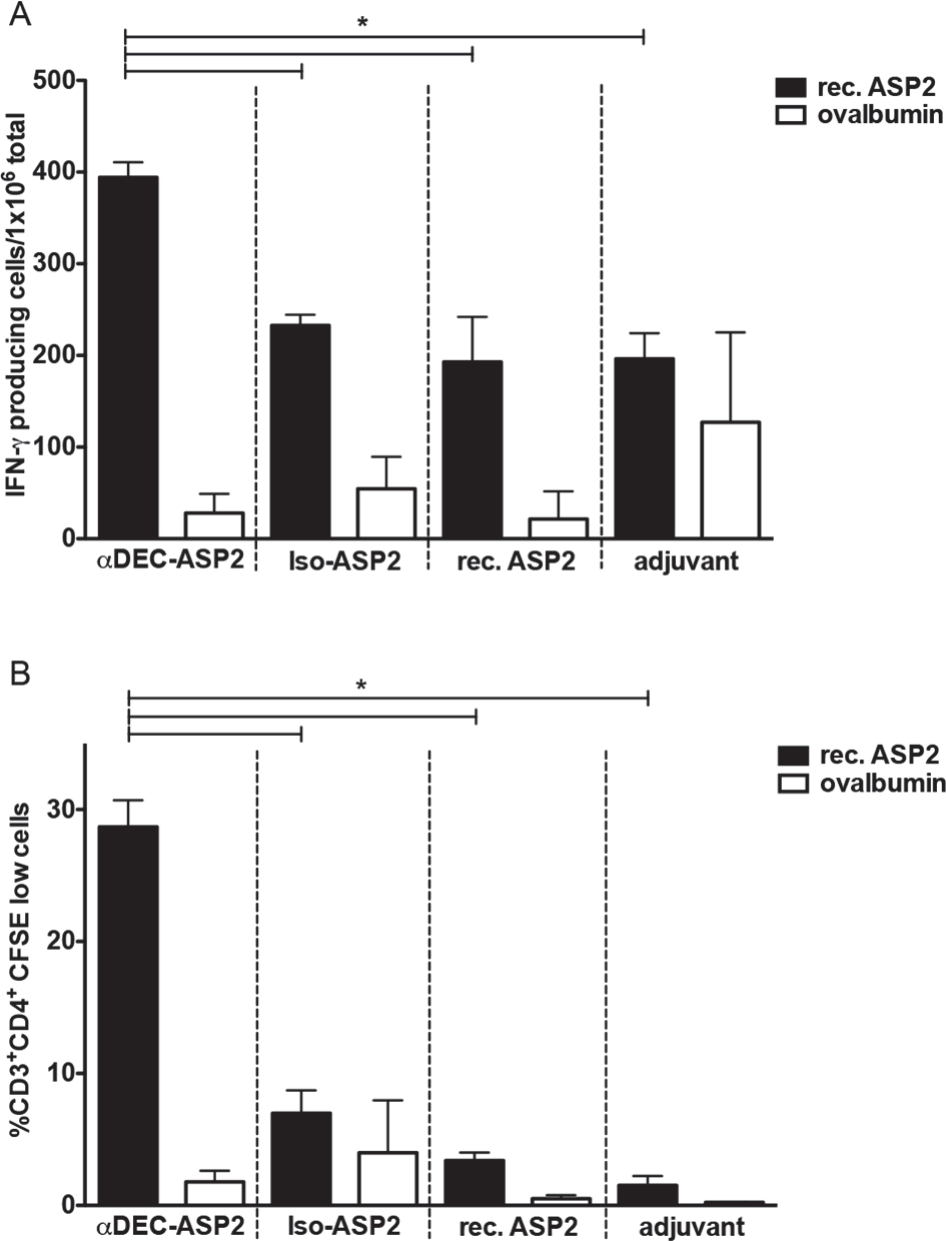
Immunization with the hybrid DEC-ASP2 mAb induces IFN-γ production and CD4^+^ T cell proliferation. BALB/c mice were immunized with two doses of the hybrid mAbs or the rec. ASP2 protein as described in materials and methods. Fifteen days after the administration of the second dose, mice were euthanized and their splenocytes were plated in the presence or absence of rec. ASP2 or ovalbumin, as a control. (A) The number of IFN-γ producing cells was detected by ELISPOT and each bar represents the mean ± SD of triplicates of pooled splenocytes. Numbers of IFN-γ producing cells in the absence of any stimulus were subtracted from the stimulated samples. (B) The percentage of CD3^+^CD4^+^ T cells that proliferated after 5 days of re-stimulation with the rec. ASP2 or ovalbumin is shown. Bars represent the mean ± SD of pooled animals performed in triplicates. Percentages of proliferating cells in the absence of any stimulus were subtracted from the stimulated samples. Experiment was analyzed by one-way ANOVA followed by the post-test HSD Tukey. * refers to p<0.05. The graph is representative of 2 (A) or 3 (B) independent experiments.

Immunizations with αDEC-ASP2 mAb were carried out with poly (I:C) as a DC maturation stimuli, as previously described [10–12,28]. Mice immunized with two doses of αDEC-ASP2 mAb showed a significant increase in the number of IFN-γ producing splenocytes when compared to mice immunized with either the isotype control, rec. ASP2 or poly I:C only (Fig. 3A, black bars). This increase was specific for ASP-2 responding cells as a smaller number of IFN-γ producing splenocytes was detected when ovalbumin was used as an unrelated antigen (Fig. 3A, white bars).

Immunization with the αDEC-ASP2 mAb resulted also in proliferation of antigen-specific CD4^+^ T cells (Fig. 3B). Spleen cells pulsed with the rec. ASP2 showed robust proliferation after the administration of two doses of αDEC-ASP2 when compared to the groups immunized with Iso-ASP2, rec. ASP2 or poly I:C (Fig. 3B, black bars). A much less significant percentage of proliferation was observed when splenocytes were pulsed with the unrelated antigen ovalbumin (Fig. 3B, white bars). The previous results lead to the conclusion that targeting the *T*. *cruzi* ASP-2 protein to the DEC205^+^ DC population promotes an increase in the number of antigen-specific IFN-γ producing cells and activation of CD4^+^ T cell proliferation.

### Identification of a CD4^+^ T-cell epitope in mice immunized with αDEC-ASP2 mAb

**Fig 4:**
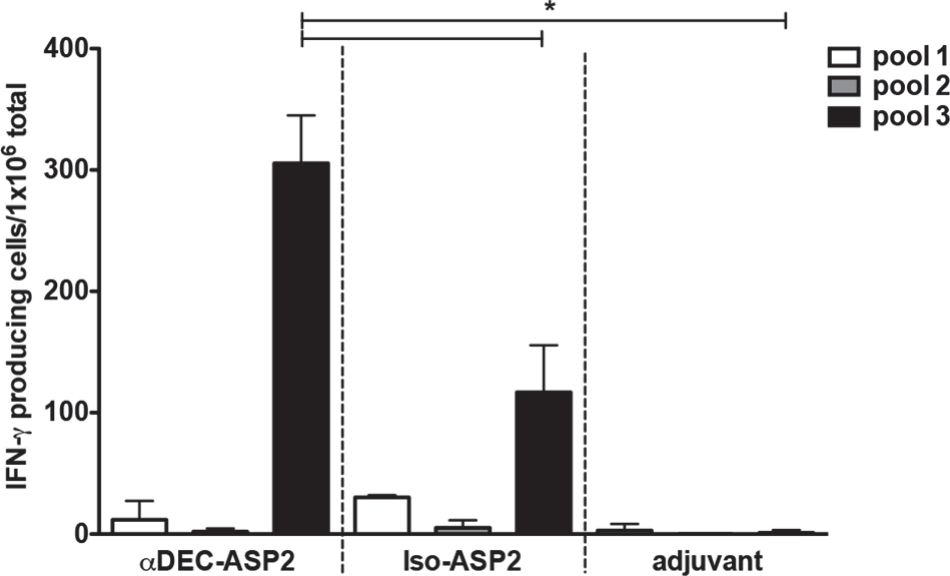
Screening of the peptide pools comprising ASP-2 amino acids 261–380. BALB/c mice were immunized with two doses of the αDEC-ASP2 or Iso-ASP2 mAbs as described in material and methods. Fifteen days after the administration of the second dose, mice were euthanized and their splenocytes were plated in the presence or absence of the three peptide pools (5 μg/mL) shown in Table 1. The number of IFN-γ producing cells was detected by ELISPOT and the bars represent the mean ± SD of pooled animals performed in triplicates. Numbers of IFN-γ producing cells in the absence of any stimulus were subtracted from the stimulated samples. Experiment was analyzed by one-way ANOVA followed by the post-test HSD Tukey. * refers to p<0.05. The graph is representative of 2 experiments.

**Fig 5:**
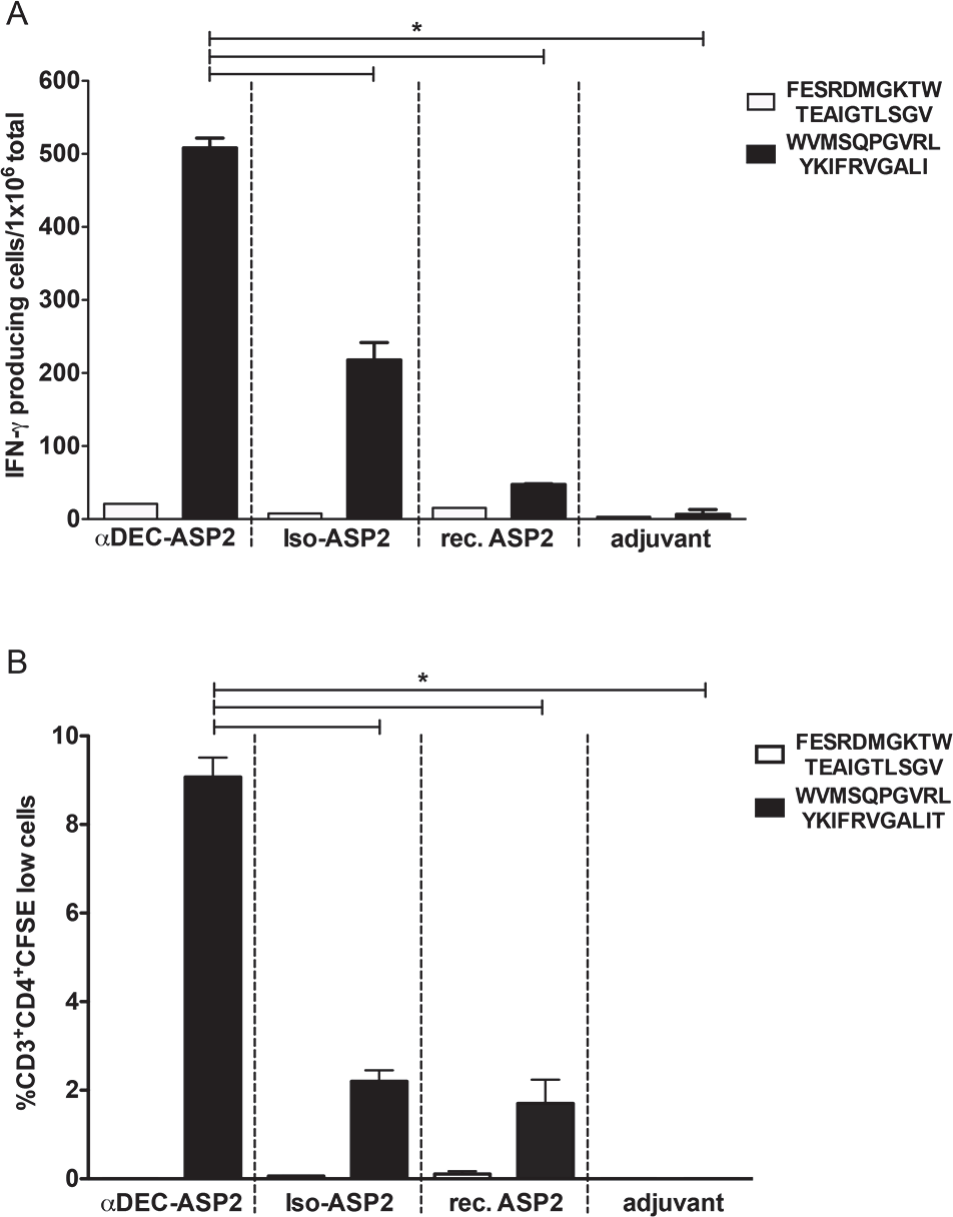
Mapping the specific T-cell epitope responsible for the response to the ASP-2 protein. BALB/c mice were immunized with two doses of the hybrid mAbs or the rec. ASP2 protein as described in materials and methods. Fifteen days after the administration of the second dose, mice were euthanized and their splenocytes were plated in the presence or absence of the peptides FESRDMGKTWTEAIGTLSGV or WVMSQPGVRLYKIFRVGALIT. (A) The number of IFN-γ producing cells was detected by ELISPOT and the bars represent the mean ± SD of pooled animals performed in triplicates. Numbers of IFN-γ producing cells in the absence of any stimulus were subtracted from the stimulated samples. (B) The percentage of CD3^+^CD4^+^ T cells that proliferated after 5 days of re-stimulation with each peptide is shown. Bars represent the mean ± SD of pooled animals performed in duplicates. Percentages of proliferating cells in the absence of any stimulus were subtracted from the stimulated samples. Experiments were analyzed by one-way ANOVA followed by the post-test HSD Tukey. * refers to p<0.05. The graphs are representative of 2 experiments.

In order to map the specific region of the ASP-2 protein responsible for the observed IFN-γ response and T cell proliferation, six peptides were synthesized and covered ASP-2 amino acids 261 to 380. The peptides were divided in 3 pools containing 2 peptides each (Table 1) and used to stimulate splenocytes derived from mice immunized with αDEC-ASP2, Iso-ASP2 or adjuvant. A specific IFN-γ response was obtained when splenocytes were stimulated with peptides present in pool 3 (Fig. 4). Mice immunized with αDEC-ASP2 presented higher numbers of IFN-γ producing cells when compared to mice immunized with Iso-ASP2. To further characterize the response, another IFN-γ ELISPOT assay was performed using the individual peptides. Peptide WVMSQPGVRLYKIFRVGALIT was identified as the responsible for the IFN-γ production (Fig. 5A). Again, a higher number of IFN-γ producing splenocytes was detected in mice immunized with αDEC-ASP2 mAb when compared to mice immunized with either Iso-ASP2 or rec. ASP2. In addition, peptide WVMSQPGVRLYKIFRVGALIT was also responsible for the strong CD4^+^ T cell proliferation observed in the splenocytes derived from mice immunized with the αDEC-ASP2 mAb (Fig. 5B). In summary, immunization of mice with αDEC-ASP2 mAb allowed us to map an ASP-2 specific CD4^+^ T cell epitope recognized by the BALB/c haplotype.

## Discussion

In this work, we took advantage of a new strategy that involves antigen targeting directly to DCs. This is accomplished by the use of DC receptor specific mAbs fused to the antigen of interest. We fused the αDEC205 mAb sequence to a portion of the ASP-2 protein expressed by the Y strain of *T*. *cruzi*. This approach led to the induction of potent immune responses against other pathogens such as Epstein-Barr virus [29], dengue virus [11], papillomavirus [30], HIV [5,10], *Plasmodium* sp. [4], *Leishmania major* [9,13], and *Yersinia pestis* [8,12]. In addition, it was also shown that αDEC205 mAb fused to antigens expressed by cancer cells could also confer immunity, and even protection, in murine models [31,32].

Although potent, this strategy relies on the construction of the fusion mAb. As the reagent is novel for each different antigen, there is always the challenge of producing an intact fusion mAb. In the present study, we successfully produced two fusion mAbs: the αDEC-ASP2 and its control Iso-ASP2. To our knowledge, this is the first time a protein larger than 60 kDa is fused to the αDEC205 mAb, as ASP-2 amino acids 82 to 694 encode a protein of approximately 65 kDa. Despite its size, the αDEC-ASP2 mAb maintained its biding capacity to either CHO cells expressing the murine DEC205 receptor constitutively or to the DC population that naturally expresses this receptor. Nonetheless, its binding curve at 10 μg/mL was slightly less effective when compared to the binding curve of the previously published αDEC-CS mAb [4]. Two hypotheses could explain this result: 1. αDEC-CS mAb is approximately 214 kDa while the molecular mass of the αDEC-ASP2 mAb is approximately 292 kDa. In this way, when we added 10 μg/mL final to each well, we were in fact adding 1,38x more αDEC-CS mAb; 2) ASP-2 was the largest protein we fused to the anti-DEC205 mAb, and this could cause conformational changes that would account for the reduction in binding. However, the preserved antigenicity of the ASP-2 protein fused to either anti-DEC205 or isotype control mAbs was evidenced by its recognition by the previously described mAb K_2_2 [25], indicating that the ASP-2 structure was maintained.

We next compared the efficiency of this targeting strategy when the antigen was targeted or not to the DEC205^+^ DC population. In accordance to data previously published in other models [4,5,11,13], ASP-2 targeting to the DEC205^+^ DC population was more efficient in eliciting IFN-γ production by splenocytes of immunized mice than its non-targeted versions (Iso-ASP2 mAb or the recombinant protein). For that reason, many efforts are focused on the use of other adjuvants and the administration of two doses of poly (I:C) was already validated by others and ourselves [10,11].

Next, we evaluated if ASP-2 targeting to the DEC205^+^ DC population was able to induce proliferation of CD4^+^ T cells. We observed robust proliferation of CD4^+^ T cells (∼30%) in the group immunized with the DEC-ASP2 mAb when compared to the Iso-ASP2 or the recombinant protein. The role of ASP-2 specific CD4^+^ T cells during *T*. *cruzi* infection was evaluated using different immunization protocols and mouse strains. Splenocytes from BALB/c mice immunized with a plasmid DNA encoding the *asp-2* gene were able to secrete IFN-γ and NO upon restimulation with the recombinant ASP-2 protein. This secretion was attributed to CD4^+^ T cells because it was inhibited after addition of an anti-CD4 monoclonal antibody [16]. The role of CD4^+^ T cells induced by immunization with the ASP-2 was further analyzed in vivo using A/Sn and C57BL/6 mice. In both strains, CD4^+^ T cell depletion in animals immunized with ASP-2 (DNA priming followed by adenovirus boosting) reduced their survival after *T*. *cruzi* infection, and IFN-γ expression by these cells was required for protection [18].

The CD4^+^ T cell proliferation observed in the mice immunized with the αDEC-ASP2 mAb prompted us to investigate if we could identify the CD4^+^ T cell epitope(s) responsible for such proliferation. For that purpose, we designed six 19–21-mer peptides that were divided into three pools containing 2 peptides each. These peptides comprised amino acids 261 to 380 of the ASP-2 protein. We decided to use this region because Araujo et al. showed, in A/Sn mouse strain, that protection was obtained when mice were immunized with a fusion protein containing this region [15]. When we analyzed IFN-γ producing cells, significantly higher numbers were obtained in mice immunized with the αDEC-ASP2 mAb and pulsed with pool 3. Separate analysis of the two peptides comprising pool 3 showed that the response was directed against the WVMSQPGVRLYKIFRVGALIT peptide.

To our knowledge, this is the first time a putative CD4^+^ T cell epitope recognized by the BALB/c haplotype is described in the ASP-2 protein sequence. In a previous study, we showed that the in vitro IFN-γ production against the recombinant ASP-2 protein elicited in mice immunized with a DNA vaccine was dependent on CD4^+^ T cells [16]. In this study, we extend those results by describing a CD4^+^ epitope that could probably be responsible for such IFN-γ production, and validate the use of DC targeting strategy to identify potential T-cell epitopes.

## Supporting Information

S1 Fig Gating strategy for the evaluation of the hybrid mAbs binding to the splenic DCs. Splenocytes were stained on ice with different mixtures of mAbs. Doublets and CD3^+^CD19^+^DX5^+^ cells were excluded from further analysis. CD11c^+^MHCII^+^ cells were gated and then separated by the expression of CD8^+^. Analysis was performed on the CD11c^+^CD8^+^and CD11c^+^CD8^-^ DCs. The numbers inside the graphs represent the percent of gated cells.
(TIF)
