# Supplementary figures and images for: Antigen Targeting to Dendritic Cells Allows the Identification of a CD4 T-Cell Epitope within an Immunodominant Trypanosoma cruzi Antigen
Source: PLoS One. 2015 Feb 13;10(2):e0117778. doi: 10.1371/journal.pone.0117778 (PMC4332658; doi:10.1371/journal.pone.0117778)

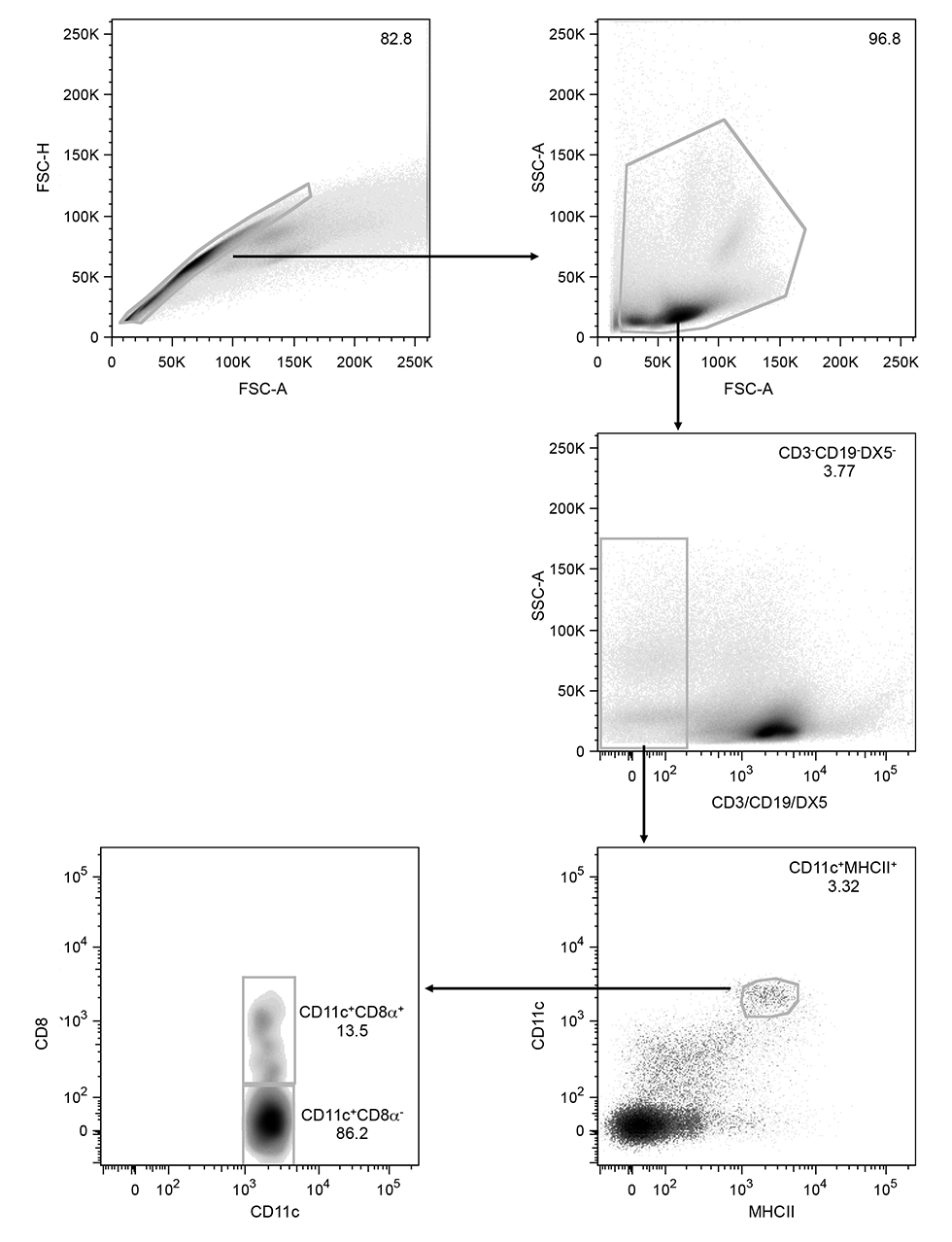

Supplement: S1 Fig — Splenocytes were stained on ice with different mixtures of mAbs. Doublets and CD3+CD19+DX5+ cells were excluded from further analysis. CD11c+MHCII+ cells were gated and then separated by the expression of CD8+. Analysis was performed on the CD11c+CD8+and CD11c+CD8- DCs. The numbers inside the graphs represent the percent of gated cells. (TIF) [file pone.0117778.s001.tif]
